# Wireless patches for continuous vital sign monitoring, symptoms and medication at the end-of-life in the palliative care unit: A prospective observational study

**DOI:** 10.1007/s10877-025-01343-6

**Published:** 2025-08-19

**Authors:** Theresa Tenge, Sebastian Reekers, Oliver Maier, Manuela Schallenburger, Yann-Nicolas Batzler, Marc Stefaniak, Jacqueline Schwartz, Alexandra Stroda, René M´Pembele, Sebastian Roth, Bahne H. Bahners, Guanqing Chen, Maximilian S. Schaefer, Christian Jung, Martin Neukirchen

**Affiliations:** 1 Interdisciplinary Centre for Palliative Medicine, Medical Faculty, University Hospital Düsseldorf, Heinrich-Heine-University Düsseldorf, Düsseldorf, Germany; 2 Department of Anaesthesiology, Medical Faculty, University Hospital Düsseldorf, Heinrich-Heine-University Düsseldorf, Düsseldorf, Germany; 3 Center for Anesthesia Research Excellence (CARE), Beth Israel Deaconess Medical Center, Harvard Medical School, Boston, MA United States of America; 4 Department of Cardiology, Pulmonology and Vascular Medicine, Medical Faculty, University Hospital Düsseldorf, Heinrich-Heine-University Düsseldorf, Düsseldorf, Germany; 5 Centre of Palliative Care and Pediatric Pain, Faculty of Medicine, Saarland University Medical Center and Saarland University, Homburg/Saar, Germany; 6 Department of Neurology, Centre for Movement Disorders and Neuromodulation, Medical Faculty, University Hospital Düsseldorf, Heinrich-Heine-University Düsseldorf, Düsseldorf, Germany

**Keywords:** Vital signs, Monitoring, physiologic, Wearable electronic devices, Palliative care, Terminal care, Death, Prospective studies, Observational study

## Abstract

Vital sign monitoring in palliative care could support symptom management and prediction, though its utility at the end-of-life remains critically discussed. This study aims to test a wireless device for continuous vital sign monitoring at the end-of-life. This prospective observational study included adult terminal care patients in the palliative care unit at a tertiary-care hospital (05/2023–03/2024). Continuous monitoring of heart rate (HR in beats/minute, bpm), respiratory rate, and temperature was conducted using VitalPatches (MediBioSense). Patient demographics, medications, and symptoms were further recorded. Linear mixed-effects models were applied for analyses. 30 patients (median age 70, 53.3% female, 90% with cancer) were included. Median patch duration was 88 h (IQR 35–153). Symptom load increased until death (*p* = 0.004), with weakness and impaired vigilance being prominent. From patch start to end, opioid use rose from 80 to 100% (*p* = 0.010), and benzodiazepine use from 50 to 80% (*p* = 0.015). All vital signs increased in daily and hourly intervals. Compared to three days prior, the HR increased as death approached: 3.70 bpm (-2 days, *p* < 0.001), 7.64 bpm (-1 day, *p* < 0.001), and 12.26 bpm (day of death, *p* < 0.001). Pain correlated with HR (*r* = 0.32, *p* < 0.001), and a 5 bpm HR increase over the previous 24 h was associated with imminent death within the following 24 h. Continuous vital sign monitoring in palliative care using VitalPatches is feasible and depicts vital sign changes at the end-of-life. A positive correlation of HR with pain was detected. These observations provide a rationale for larger studies investigating their relevance for life time prediction as well as symptom detection and management.

**Supplementary Information:**

The online version contains supplementary material available at 10.1007/s10877-025-01343-6.

## Introduction

Palliative care aims to improve the quality of life for patients facing serious, life-limiting illnesses, by focusing on physical, psychosocial and spiritual symptom relief and support for both patients and their families [1]. Vital sign monitoring is a common clinical practice, yet its role in palliative care remains a subject of ongoing debate [2, 3]. While vital signs may offer valuable data, their collection in a palliative care setting may pose more harm than benefit, given the potential for disturbance or discomfort to the patient, family members, and care providers [2]. Despite these concerns, vital signs can serve as an objective parameter to assist in predicting the need for supportive measures and imminence of death, supporting clinicians’ clinical judgment, and enhancing communication with patients and families about prognosis and care planning [2]. Therefore, studies investigated different devices for the collection of vital sign data [4–8].

Previous studies have explored the use of serial vital sign monitoring for end-of-life care [9–13]. However, the investigation of continuous monitoring has remained sparse [14, 15]. Continuous monitoring could provide more detailed and real-time data including trends, potentially enhancing prognostication.

This study aims to: (1) assess the feasibility of continuous vital sign monitoring using wearable patches in the palliative care unit, and (2) analyse vital sign changes at the end-of-life and examine how these changes correlate with symptoms and influence medication use, in order to identify patterns that may enhance clinical prognostication, symptom detection, and symptom management.

## Methods

### Study design & population

This prospective observational study was reviewed and approved by the local ethics committee (protocol number: 2022 − 1981_1, March 18th 2023). We recruited patients aged 18 years or older who were hospitalized in the palliative care unit at a tertiary-care centre in Germany between May 2023 and March 2024. We included patients in terminal care whose life expectancy was estimated to be no more than a few days by the multi-professional palliative care team, and written informed consent was obtained. This manuscript adheres to the Strengthening the Reporting of Observational Studies in Epidemiology (STROBE) guidelines [16].

### Data collection

After inclusion, we set up a continuous, wireless, wearable vital sign monitor patch (VitalPatch, MediBioSense, Doncaster, United Kingdom) on the patient’s left chest as outlined by the manufacturer. The VitalPatch biosensor includes two electrocardiography electrodes, a triaxial accelerometer, and a thermistor. The patch detects the heart rate (HR in beats/minute), respiratory rate (RR in breaths/minute), body temperature (Temp in °Celsius) every four seconds, and has been validated in previous studies [17, 18]. Data were sent via Bluetooth to mobile phones, which uploaded the data over cellular networks to the device’s online platform. Subsequently, data were extracted as hourly mean values, after cleaning the HR for values below 30 and greater than 155 beats/minute. Clinical measurements were regularly performed to confirm the correctness of the detected vital signs. We also collected general clinical data from the patient’s medical records, including demographics, comorbidities, medications at start and end of patch application, as well as symptom assessments in the patient’s chart which are documented three times a day in each nursing shift. Symptom assessment in the palliative care unit was based on the Palliative Symptom Burden Score (PSBS), which includes ten items and a symptom intensity scale ranging from 0 to 4 [19, 20]. Further, the documented time of death in the notes was obtained to compare time differences with the patch data.

### Data analysis

To assess the symptom load, a total score was calculated by summing up the individual symptom intensities [20]. For a total of 10 symptoms (vigilance, confusion, anxiety, sweating, weakness, nausea, vomiting, dyspnoea, cough and itching), intensities ranged from 0 (none) to 4 (severe). A detailed description of each symptom at each level is provided in the *Supplementary Information*. Constipation was assessed as a binary variable (1 = yes, 0 = no) and pain using the numeric rating scale (NRS) ranging from 0 (no pain) to 10 (worst pain imaginable) [21]. A linear mixed-effects model to study the association between time until death and symptom load with the individual case as the random effects and adjustment for age, sex and body mass index as in previous analyses was carried out [15]. Differences in the medication at the start and end of patch application were investigated by the mean standardized difference and chi-square tests. Mean standardized differences of 0.2, 0.5, and 0.8 are considered small, medium, and large, respectively [22]. For vital sign analyses, adjusted linear mixed-effects models were applied to explore the association between time before death and vital signs with the individual case as the random effects and adjustment for age, sex and body mass index. First, we assessed the association between days before death and HR, RR and Temp, respectively. Data from the third day before death was used as the reference, as previously published [12]. Subsequently, we examined the association between hours before death and the respective vital signs, using 72 h before death as the reference. We also assessed the association between an increase in HR of five or ten beats per minute over the last 24 h and imminent death within the next 24 h using logistic regression. Sensitivity analyses were conducted using time in days and hours as continuous variables and excluding data from the last two hours before death as in previous literature [15]. We applied likelihood ratio tests to confirm that the inclusion of the random effect significantly improved the model fit. With an exploratory intent, for each shift before death the mean vital signs were individually correlated with the symptom intensities using Spearman correlation applying Bonferroni [23]. A *p*-value < 0.05 was considered statistically significant. We obtained standardized coefficients or adjusted odds ratios (OR) and two-sided 95% confidence intervals (CI). Analyses and data visualization were conducted using STATA 18.0 (StataCorp LLC, College Station, TX, USA).

## Results

### Study cohort and patch data

For this study, 35 patients were recruited and 30 patients included in the final analysis. One patient removed the patch during hyperactive delirium, for the other four patients technical and connection problems occurred and led to missing data. The median age was 70 (interquartile range [IQR] 61–81) years, 53.3% of patients were female. The median body mass index was 23.9 (IQR 20.9–26.8). At inclusion, the functional status of patients was partially (26.7%, *n* = 8) or fully (73.3%, *n* = 22) dependent. The comorbidities of the participants are summarized in Table 1, showing that 90% of patients suffered from cancer.

**Table 1:** Patient comorbidities data are presented as frequency and prevalence (%)

Comorbidities	Yes
Arterial hypertension	16 (53.3%)
NYHA	
I	0 (0.0%)
II	2 (6.7%)
III	1 (3.3%)
IV	1 (3.3%)
Pulmonary hypertension	0 (0.0%)
Diabetes mellitus	5 (16.7%)
Chronic vascular disease	0 (0.0%)
Peripheral vascular disease	1 (3.3%)
Chronic obstructive lung disease	4 (13.3%)
Smoking	13 (43.3%)
Dialysis	0 (0.0%)
Immunogenic therapy	0 (0.0%)
Condition after TIA/Stroke	3 (10.0%)
Condition after cardiac decompensation (1 year prior)	3 (10.0%)
Condition after myocardial infarction	3 (10.0%)
Pacemaker/ICD	0 (0.0%)
Condition after CPR	0 (0.0%)
Dementia	1 (3.3%)
Cancer	27 (90.0%)
Metastasis	19 (63.3%)

NYHA, New York Heart Association. TIA, Transient Ischemic Attack

ICD, Implantable Cardioverter-Defibrillator. CPR, Cardiopulmonary Resuscitation

Almost all patients were transferred to the palliative care unit at the day of hospital admission (one after 16, another after 41 inpatient days hospitalized). The duration from unit admission until patch start was 7 days (IQR 2–10). Median patch duration was 88 hours (IQR 35–153) and 3 days (IQR 1–6), respectively. All patients died in the unit, the time difference between the detected time of death from the patches compared to the clinical notes was 3 minutes (IQR 0–7).

### Symptoms

**Fig. 1:**
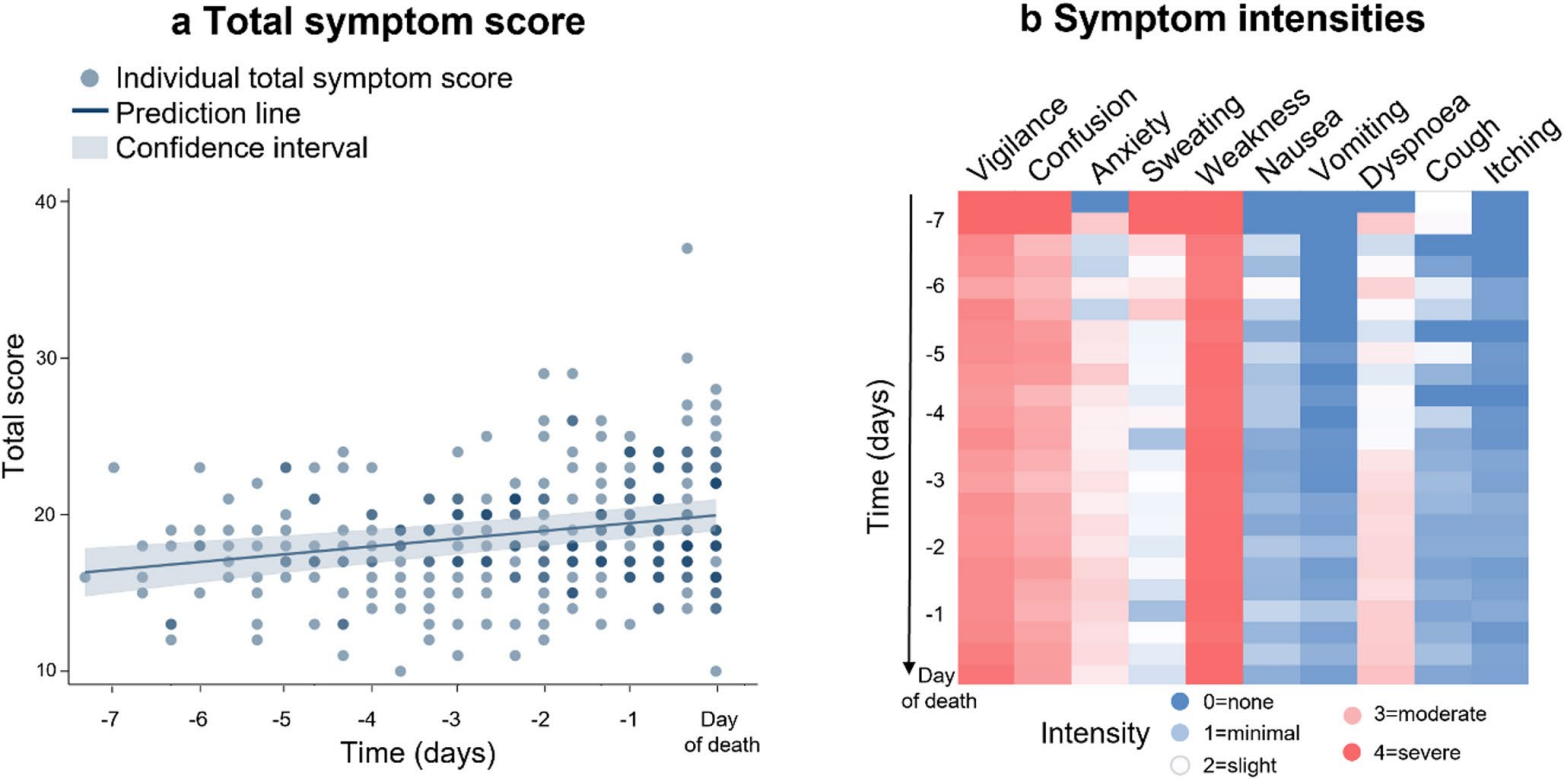
Symptoms over time. The total symptom score **a** shows an increasing trend of the cumulative symptom load and intensity at each shift before death. The individual symptom intensities differ **b.**

Overall, symptom load increased closer to death (0.12, 95% CI 0.04 to 0.19, *p* = 0.004; Fig. 1a). For the last shift before death, the median total score was 19 (IQR 16–23). Detailed information is provided in the *Supplementary Information*. High symptom intensities were observed in weakness, impaired vigilance and confusion (Fig. 1b). For pain assessment, mean pain NRS values throughout the observation period were between 2.0 (standard deviation [SD] 1.6) and 3.8 (SD 1.5). Constipation was present in 58.6% (*n* = 17) of patients.

### Medication

At the start of patch use, around half of the patients received non-opioids and 80% opioids. This prevalence changed towards the end of patch application (10% and 100%, respectively). Additionally, benzodiazepines were often prescribed at time of death (80% vs. 50%, *p* = 0.015). Use of other medications, such as antidepressants, antiemetics, laxatives, intravenous infusions, antibiotic and antithrombotic agents, were reduced (Table 2).

**Table 2:** Prevalence of medication use at the start and end of patch application. Data are presented as frequency and prevalence (%). Mean standardized differences of 0.2, 0.5, and 0.8 are considered small, medium, and large, respectively. *P*-values were derived from chi-square tests

Check	Start *n* = 30	End *n* = 30	Mean standardized difference	*P*-value
Non-opioid analgesics	16 (53%)	3 (10%)	1.035	< 0.001
Opioids	24 (80%)	30 (100%)	−0.695	0.010
Antidepressants	7 (23%)	0 (0%)	0.767	0.005
Anticonvulsants	5 (17%)	3 (10%)	0.194	0.45
Antiemetic agents	16 (53%)	6 (20%)	0.725	0.007
Laxatives	3 (10%)	0 (0%)	0.463	0.076
Other analgesics	2 (7%)	0 (0%)	0.372	0.15
Benzodiazepines	15 (50%)	24 (80%)	−0.651	0.015
Intravenous infusions	7 (23%)	2 (7%)	0.472	0.071
Parenteral nutrition	3 (10%)	1 (3%)	0.265	0.30
Enteral nutrition	0 (0%)	0 (0%)		
Antihypertensive agents	3 (10%)	0 (0%)	0.463	0.076
Antiarrhythmic agents	1 (3%)	0 (0%)	0.258	0.31
Diuretics	2 (7%)	0 (0%)	0.372	0.15
Anticholinergics	3 (10%)	7 (23%)	−0.358	0.17
Antibiotics	3 (10%)	0 (0%)	0.463	0.076
Antithrombotic/Anticoagulant agents	5 (17%)	0 (0%)	0.622	0.020
Proton pump inhibitors	7 (23%)	1 (3%)	0.605	0.023

### Vital signs

**Fig. 2:**
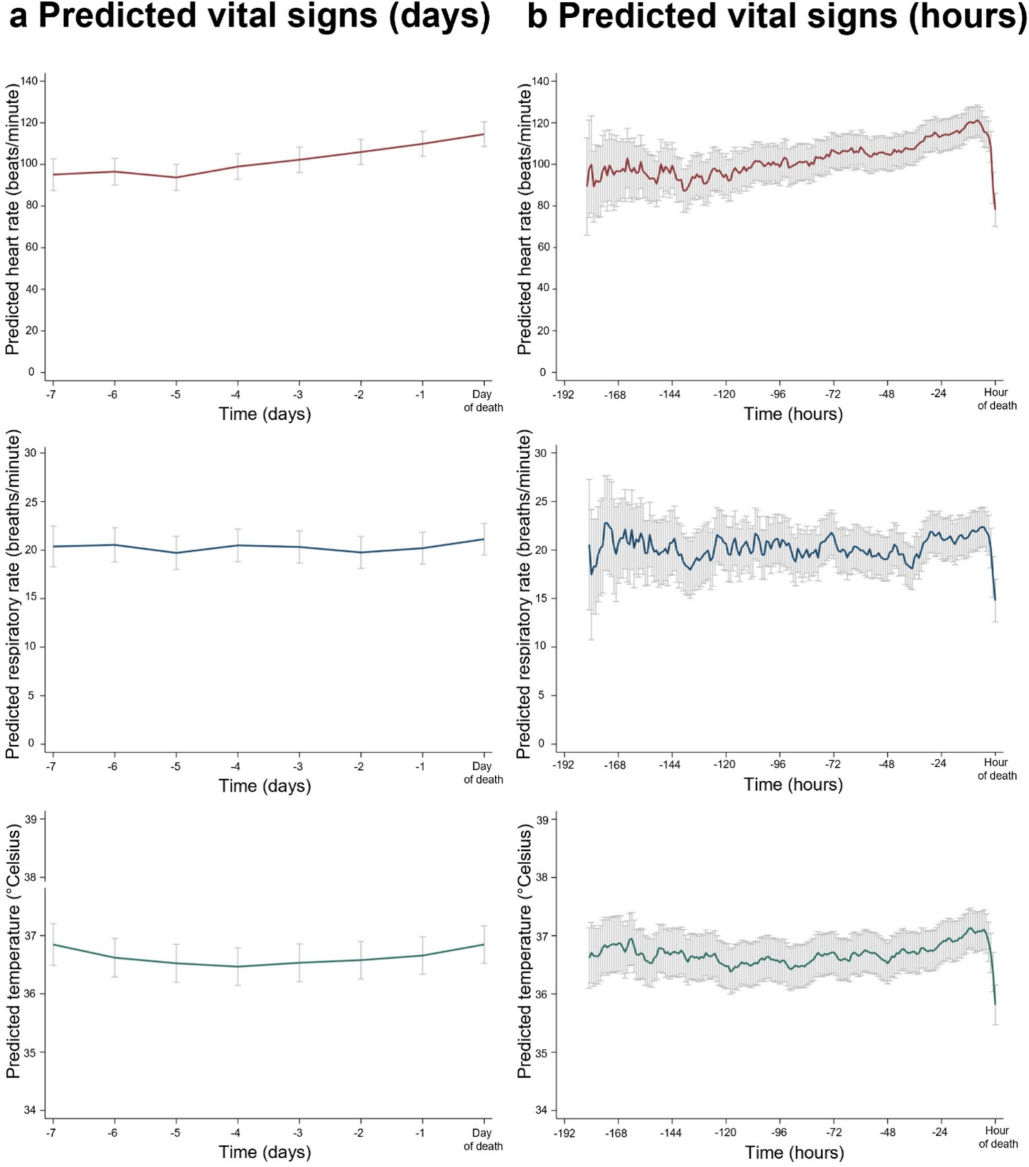
Vital signs over time. The predicted vital signs heart rate (red), respiratory rate (blue) and temperature (green) are shown for each day **a** and hour **b** before death

Vital sign monitoring in all included patients was unproblematic and well tolerated. No complaints from patients, relatives or staff members occurred. In the primary analysis (Fig. 2a), compared to three days prior to death, the HR was significantly lower before (−7 days: −7.13, 95% CI −12.07 to −2.19, *p* = 0.005; −6 days: −5.73, 95% CI −8.60 to −2.84, *p* < 0.001; −5 days: −8.52, 95% CI −10.94 to −6.10, *p* < 0.001; −4 days: −3.28, 95% CI −5.43 to −1.13, *p* = 0.003) and higher afterwards (−2 days: 3.70, 95% CI 1.81 to 5.59, *p* < 0.001; −1 day: 7.64, 95% CI 5.79 to 9.48, *p* < 0.001; day of death: 12.26, 95% CI 10.42 to 14.11, *p* < 0.001). For the RR, compared to three days prior to death, the RR decreased on the following day and increased on the day of death (−2 day: −0.70, 95% CI −1.25 to −0.16, *p* = 0.012; day of death: 0.77, 95% CI 0.24 to 1.31, *p* = 0.005). The temperature was higher compared to 3 days prior before, on day 7 before death (−7 day: 0.33, 95% CI 0.17 to 0.50, *p* < 0.001) and increased again on the last two days of life (−1 day: 0.15, 95% CI 0.09 to 0.21, *p* < 0.001; day of death: 0.34, 95% CI 0.30 to 0.42, *p* < 0.001). When comparing the vital signs to those recorded 72 h before death, the same trends observed in the primary analyses were evident (see *Supplementary Material*). Figure 2b illustrates the predicted vital signs at each hour before death. An increasing HR of more than five beats per minute compared to the value 24 h prior was significantly associated with imminent death within the following 24 h (OR, 1.86; 95% CI 1.56–2.22; *p* < 0.001). This association was magnified for an increased HR of ten beats per minute (OR, 2.29; 95% CI 1.88–2.78; *p* < 0.001).

Sensitivity analyses including time in days and hours as continuous variables revealed an increase in all vital signs (HR hourly: 0.15, 95% CI 0.13 to 0.16, *p* < 0.001; HR daily: 3.58, 95% CI 3.25 to 3.91, *p* < 0.001; RR hourly: 0.005 95% CI 0.001 to 0.007, *p* = 0.004; RR daily: 0.11, 95% CI 0.02 to 0.21, *p* = 0.015; Temp hourly: 0.002, 95% CI 0.002 to 0.003, *p* < 0.001; Temp daily: 0.05, 95% CI 0.04 to 0.06, *p* < 0.001). Excluding the last two hours before death yielded confirmatory results.

### Correlation between symptoms and vital signs

**Fig. 3:**
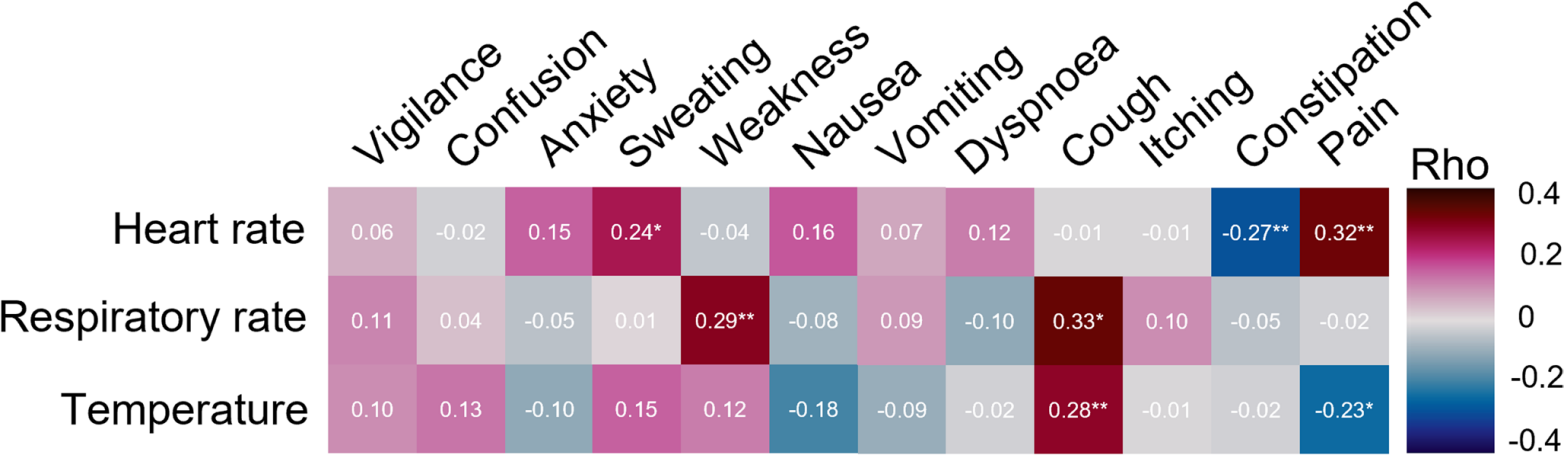
Spearman correlations between assessed symptom intensities and the mean vital sign at each shift statistically significant correlations are marked with * for *p* < 0.05 and ** for *p* < 0.001

Correlation analyses were conducted to examine the relationship between the assessed symptom intensities and the mean vital signs at each shift (Fig. 3). Pain and sweating showed a positive correlation with HR, while constipation exhibited a negative correlation. Cough and weakness were positively correlated with RR, with cough also correlating with elevated body temperature. Additionally, pain was inversely correlated with temperature.

## Discussion

### Main findings

In this prospective single-centre observational cohort study conducted in the palliative care unit, the use of continuous vital sign monitoring with a wearable patch was feasible – supporting and extending previous findings from other non-critical care settings [24]. No complaints were reported. However, in one case, the patch was removed by a patient experiencing delirium, leading to their exclusion from the final analysis. Changes in symptoms, including an overall increase, and adaptations in medication at the end-of-life were observed. Vital signs, including HR, RR and Temp, showed an increasing trend towards death. For HR, a marked increase was observed in the last two days before death, and an increase of five or ten beats per minute within the final 24 h was associated with imminent death. HR was positively correlated with pain intensity. Changes in RR and Temp were minimal.

### What this study adds

The scoping review by Power et al. summarized studies on vital sign monitoring in palliative care, highlighting the limited data on serial or continuous measurements. Our findings are consistent with previous research on continuous vital sign monitoring [15]. A study using non-wearable devices placed under the mattress of participants, with a mean measurement period of 10 days, reported significant increases in HR (1.51 beats/min, 95% CI 1.50 to 1.52) and RR (0.27 beats/min, 95% CI 0.27 to 0.28). However, the study population differed, as it included only cancer patients with a mean body mass index of 18.6 and a mean age of 76.7 years [15]. In our study, 90% also had a cancer diagnosis but the cohort was younger and had a lower body mass index.

Another study, which monitored vital signs every twelve hours in non-cancer patients, demonstrated that systolic blood pressure, diastolic blood pressure, and oxygen saturation significantly decreased from day − 3 to death [12]. The increase in HR observed in our study during this period may be partially explained by these findings, as a decrease in blood pressure could trigger an increase in HR to maintain organ perfusion.

The study by Bruera included measurements of HR, blood pressure, RR, oxygen saturation, and Temp [13]. In this cohort of 357 patients, vital signs were measured twice daily from admission until death or discharge. The data support the proposed hypothesis, as systolic blood pressure, diastolic blood pressure, and oxygen saturation significantly decreased in the final three days of life. Temperature slightly increased, while HR and RR remained stable in the last three days. Notably, 55% of patients had an HR greater than 100 beats per minute in the last two days of life, which aligns with the relatively high values observed in our study.

Overall, based on the minimal changes in vital signs at the end-of-life, a comment on the aforementioned study concluded that vital signs alone are insufficient for clinicians to accurately prognosticate the timing of death. The author states that “the benefit of routine collection of vital signs of patients whose goal is comfort may not outweigh the burden of their collection to patients, families, and staff. The decision to collect vital signs should be individualized” [3]. However, the use of non-burdensome wireless patches, as employed in our study, enables continuous monitoring, and the analysis of vital sign trends over the preceding 24 h revealed an association with imminent death.

The prevalent symptoms observed in our study align with previous data on symptoms and distress at the end-of-life [25–27]. Electronic symptom monitoring in home-based palliative care has shown potential in previous studies, but could be supported by additional vital sign monitoring [28]. Our data indicate correlations between symptom intensities and vital signs. Further studies are needed to explore whether the observed increase in HR is driven by pain, lower blood pressures or represents an independent phenomenon. Additionally, we observed changes in medication between the start and end of patch application, suggesting that care teams adjust treatment in response to evolving symptoms and needs. To address common potentially inappropriate medication in terminal patients, the “Appropriate medication use in Dutch terminal care: study protocol of a multicentre stepped-wedge cluster randomized controlled trial (the AMUSE study)” is currently conducted [29].

Ultimately, a study examining the efficacy of the 3-day surprise question (Would I be surprised if this patient died in the next 3 days?) in predicting the prognosis of advanced cancer patients nearing death found that physician performance yielded a sensitivity of 94.3% and specificity 92.7% [30]. Vital sign monitoring which is not perceived burdensome, might support clinician’s clinical prognostication, but only marginal differences were observed needing further investigations for optimized individual prediction.

### Strengths and limitations of the study

Aside from its strength in providing broad and granular real-world data on terminal patients, this study has several limitations. The single-centre design limits its generalizability, and selection bias may have occurred, as informed consent was required, excluding patients without capacity or legal representatives to provide consent. Symptom assessment was performed using the local standard, the PSBS, which was heuristically developed by clinical experts, is not widely distributed across centres and countries, and requires further adjustments based on previous evaluation [19]. Vital signs only included HR, RR and Temp, but blood pressure and oxygen saturation data are missing. HR values were intermittently compared with clinical measurements; however, no structured protocol or statistical correlation analysis was conducted. The patches also aimed to record the patient’s position. However, these were infrequent and unreliable. Furthermore, no questionnaire-based or qualitative assessment of the feasibility was performed.

In summary, this prospective observational study examined the feasibility of continuous vital sign monitoring using a wearable patch in the palliative care unit. It explored changes in vital signs, symptoms, and medications in terminal patients. We identified an increase in HR and symptoms, medication was adjusted and reduced at the end-of-life.

## Supplementary Information

Below is the link to the electronic supplementary material.

Supplementary Material 1

Supplementary Material 2

## Data Availability

No datasets were generated or analysed during the current study.

## Declarations

### Data management and sharing

The data related to this study is maintained and managed according to organizational guidelines and ethical regulations. In the interest of patient confidentiality and anonymity, this information will not be made publicly available. Requests for further information can be directed to the corresponding author.
