# Supplementary material for: Wireless patches for continuous vital sign monitoring, symptoms and medication at the end-of-life in the palliative care unit: A prospective observational study
Source: J Clin Monit Comput. 2025 Aug 19;40(2):547–55. doi: 10.1007/s10877-025-01343-6 (PMC13053415; doi:10.1007/s10877-025-01343-6)
Supplement: Supplementary file 2 — Supplementary Material 2 [file 10877_2025_1343_MOESM2_ESM.docx]

***Supplementary Information***

**Wireless patches for continuous vital sign monitoring, symptoms and medication at the end-of-life in the palliative care unit: A prospective observational study**

Theresa Tenge^1,2,3^, Sebastian Reekers^1^, Oliver Maier^4^, Manuela Schallenburger^1^, Yann-Nicolas Batzler^5^, Marc Stefaniak^1^, Jacqueline Schwartz^1^, Alexandra Stroda^2^, René M´Pembele^2^, Sebastian Roth^2^, Bahne H. Bahners^6^, Guanqing Chen^3^, Maximilian S. Schaefer^2,3^, Christian Jung^4^, Martin Neukirchen^1,2^

^1^Interdisciplinary Centre for Palliative Medicine, Medical Faculty, University Hospital Düsseldorf, Heinrich-Heine-University Düsseldorf, Düsseldorf, Germany

^2^Department of Anaesthesiology, Medical Faculty, University Hospital Düsseldorf, Heinrich-Heine-University Düsseldorf, Düsseldorf, Germany

^3^Center for Anesthesia Research Excellence (CARE), Beth Israel Deaconess Medical Center, Harvard Medical School, Boston, Massachusetts, United States of America

^4^Department of Cardiology, Pulmonology and Vascular Medicine, Medical Faculty, University Hospital Düsseldorf, Heinrich-Heine-University Düsseldorf, Düsseldorf, Germany

^5^Centre of Palliative Care and Pediatric Pain, Saarland University Medical Center and Saarland University Faculty of Medicine, Homburg/Saar, Germany
Centre of Palliative Care and Pediatric Pain, Saarland University Medical Center and Saarland University Faculty of Medicine, Homburg/Saar, Germany

^6^Department of Neurology, Centre for Movement Disorders and Neuromodulation, Medical Faculty, University Hospital Düsseldorf, Heinrich-Heine-University Düsseldorf, Düsseldorf, Germany

***Correspondence to**: Theresa Tenge, MD

Department of Anaesthesiology, University Hospital Düsseldorf

Moorenstraße 5, 40225 Düsseldorf, Germany

E-Mail: theresa.tenge@med.uni-duesseldorf.de

ORCID: 0000-0001-9529-2461

**eTable 1** Individual symptom intensity descriptions

| **Symptom** | 0 | 1 | 2 | 3 | 4 |
| --- | --- | --- | --- | --- | --- |
| **Vigilance** | no impairment | daytime fatigue | occasional sleep phases during the day | predominant sleep phases during the day | somnolence |
| **Confusion** | no impairment | patient too weak to actively orient themselves in time and place | patient suffers from short-term disorientation | patient feels significantly impaired, reacts depressively | severely disoriented and helpless |
| **Anxiety** | no impairment | occasional, patient can name the cause | occurring more frequently, attention required | episodic despite continuous medication | severe restlessness, panic, or suicidal tendencies |
| **Sweating** | no impairment | occasional after physical exertion | sweating episodes with a feeling of weakness | heavy sweating (change of clothing needed) | persistent, recurrent sweating |
| **Weakness** | no impairment | normal daily routine possible with exertion | normal daily routine possible with exertion and rest breaks | patient requires help with daily tasks | predominantly dependent on care |
| **Nausea** | no impairment | temporary, in connection with loss of appetite | occasional but multiple times a day | strong impairment, gagging and vomiting urges | lasting for hours or constant nausea |
| **Vomiting** | no impairment | single episode after food or medication intake | spontaneous vomiting | multiple episodes of vomiting per shift | tormenting vomiting or constant nausea |
| **Dyspnoea** | no impairment | occurring under exertion | occurring under minimal exertion | resting dyspnea | shortness of breath with episodic anxiety, medication required |
| **Cough** | no impairment | productive cough | dry cough | constant cough irritation, sleep disturbance | tormenting cough irritation, pain or fear of choking |
| **Itching** | no impairment | occasional | scratch defects of the skin | tormenting itching, medication required | tormenting itching despite therapy |

**eTable 2** Total symptom score before death

| **Day** | **Shift** | **n** | **Mean ± SD** | **Median (IQR)** |
| --- | --- | --- | --- | --- |
|  | -22 | 1 | 16 ± 0 | 16 (16-16) |
| -7 | -21 | 1 | 23 ± 0 | 23 (23-23) |
|  | -20 | 3 | 16.33 ± 1.53 | 16 (15-18) |
|  | -19 | 5 | 15 ± 3.24 | 13 (13-18) |
| -6 | -18 | 5 | 18.6 ± 2.88 | 18 (18-19) |
|  | -17 | 5 | 18.2 ± 1.92 | 18 (17-19) |
|  | -16 | 7 | 16.43 ± 3.51 | 16 (13-19) |
| -5 | -15 | 8 | 19.13 ± 2.70 | 18.5 (17-21.5) |
|  | -14 | 9 | 18.11 ± 3.06 | 17 (17-21) |
|  | -13 | 10 | 17 ± 4.24 | 17 (13-19) |
| -4 | -12 | 10 | 17.9 ± 2.69 | 17.5 (16-20) |
|  | -11 | 11 | 16.64 ± 2.77 | 18 (15-19) |
|  | -10 | 13 | 17.08 ± 2.93 | 17 (15-19) |
| -3 | -9 | 15 | 17.93 ± 3.35 | 18 (16-20) |
|  | -8 | 14 | 18.43 ± 2.95 | 18.5 (17-20) |
|  | -7 | 16 | 18.38 ± 3.16 | 19 (16.5-21) |
| -2 | -6 | 18 | 18.83 ± 4.58 | 18 (16-21) |
|  | -5 | 19 | 19.26 ± 4.34 | 18 (16-23) |
|  | -4 | 20 | 19.15 ± 3.22 | 19 (17-20.5) |
| -1 | -3 | 23 | 19.65 ± 3.17 | 19 (17-22) |
|  | -2 | 25 | 19.28 ± 3.09 | 19 (17-21) |
|  | -1 | 28 | 20.43 ± 5.01 | 18.5 (17-23) |
| Day of death | 0 | 30 | 19.60 ± 4.37 | 19 (16-23) |

**eTable 3** The association between time before death in hours and heart rate in beats per minute.

Output following linear mixed effects model with hour 72 before death as the reference.

| **Hour** | **Coefficient** | **Standard Error** | **p-value** | **95 % CI (lower)** | **95% CI (upper)** |
| --- | --- | --- | --- | --- | --- |
| -183 | -17.73288 | 11.94228 | 0.138 | -41.13932 | 5.673559 |
| -182 | -17.27481 | 11.94228 | 0.148 | -40.68125 | 6.131631 |
| -181 | -8.66793 | 11.94228 | 0.468 | -32.07437 | 14.73851 |
| -180 | -6.661697 | 11.94228 | 0.577 | -30.06813 | 16.74474 |
| -179 | -17.25038 | 8.7206 | 0.048 | -34.34244 | -.15832 |
| -178 | -14.25945 | 8.7206 | 0.102 | -31.35151 | 2.832611 |
| -177 | -15.01314 | 8.7206 | 0.085 | -32.10521 | 2.078919 |
| -176 | -14.66936 | 8.7206 | 0.093 | -31.76142 | 2.422701 |
| -175 | -9.738721 | 8.7206 | 0.264 | -26.83078 | 7.353341 |
| -174 | -7.499647 | 8.7206 | 0.390 | -24.59171 | 9.592415 |
| -173 | -11.93127 | 8.7206 | 0.171 | -29.02333 | 5.160796 |
| -172 | -7.008002 | 8.7206 | 0.422 | -24.10006 | 10.08406 |
| -171 | -9.17644 | 8.7206 | 0.293 | -26.2685 | 7.915622 |
| -170 | -11.59669 | 8.7206 | 0.184 | -28.68875 | 5.495372 |
| -169 | -9.802989 | 8.7206 | 0.261 | -26.89505 | 7.289074 |
| -168 | -9.877437 | 7.338666 | 0.178 | -24.26096 | 4.506085 |
| -167 | -9.99581 | 7.338666 | 0.173 | -24.37933 | 4.387712 |
| -166 | -8.6087 | 7.338666 | 0.241 | -22.99222 | 5.774822 |
| -165 | -10.0469 | 7.338666 | 0.171 | -24.43042 | 4.336623 |
| -164 | -3.580933 | 7.338666 | 0.626 | -17.96445 | 10.80259 |
| -163 | -8.534484 | 7.338666 | 0.245 | -22.91801 | 5.849038 |
| -162 | -10.21302 | 7.338666 | 0.164 | -24.59654 | 4.170501 |
| -161 | -8.193796 | 7.338666 | 0.264 | -22.57732 | 6.189726 |
| -160 | -10.74496 | 6.541158 | 0.100 | -23.56539 | 2.075475 |
| -159 | -10.33156 | 6.541158 | 0.114 | -23.152 | 2.488872 |
| -158 | -5.346725 | 6.541158 | 0.414 | -18.16716 | 7.47371 |
| -157 | -9.974132 | 6.541158 | 0.127 | -22.79457 | 2.846303 |
| -156 | -10.8655 | 6.009525 | 0.071 | -22.64395 | .9129563 |
| -155 | -12.20786 | 6.009525 | 0.042 | -23.98631 | -.4294089 |
| -154 | -13.5779 | 6.009525 | 0.024 | -25.35635 | -1.799443 |
| -153 | -13.20817 | 5.627262 | 0.019 | -24.2374 | -2.178942 |
| -152 | -13.47122 | 5.627262 | 0.017 | -24.50045 | -2.441988 |
| -151 | -15.94196 | 5.627262 | 0.005 | -26.97119 | -4.912729 |
| -150 | -12.14144 | 6.00709 | 0.043 | -23.91512 | -.3677641 |
| -149 | -6.336577 | 6.00709 | 0.291 | -18.11026 | 5.437102 |
| -148 | -10.34957 | 6.00709 | 0.085 | -22.12325 | 1.424109 |
| -147 | -7.66467 | 6.00709 | 0.202 | -19.43835 | 4.109009 |
| -146 | -8.847274 | 6.00709 | 0.141 | -20.62095 | 2.926405 |
| -145 | -10.88224 | 6.00709 | 0.070 | -22.65592 | .891441 |
| -144 | -7.070684 | 5.627262 | 0.209 | -18.09992 | 3.958547 |
| -143 | -10.18786 | 5.627262 | 0.070 | -21.21709 | .8413677 |
| -142 | -13.85564 | 5.627262 | 0.014 | -24.88487 | -2.82641 |
| -141 | -14.27616 | 5.337333 | 0.007 | -24.73714 | -3.815179 |
| -140 | -13.91121 | 5.337333 | 0.009 | -24.37219 | -3.450225 |
| -139 | -19.22536 | 5.337333 | 0.000 | -29.68634 | -8.764382 |
| -138 | -19.16998 | 5.337333 | 0.000 | -29.63096 | -8.709 |
| -137 | -17.60242 | 5.337333 | 0.001 | -28.0634 | -7.141436 |
| -136 | -15.3959 | 5.337333 | 0.004 | -25.85688 | -4.934923 |
| -135 | -12.54341 | 5.337333 | 0.019 | -23.00439 | -2.082429 |
| -134 | -13.24813 | 5.337333 | 0.013 | -23.70911 | -2.787146 |
| -133 | -11.31602 | 5.337333 | 0.034 | -21.777 | -.8550362 |
| -132 | -12.76945 | 5.337333 | 0.017 | -23.23043 | -2.30847 |
| -131 | -12.11051 | 5.337333 | 0.023 | -22.57149 | -1.649529 |
| -130 | -15.73323 | 5.337333 | 0.003 | -26.19421 | -5.272253 |
| -129 | -15.79075 | 5.337333 | 0.003 | -26.25173 | -5.329769 |
| -128 | -14.55379 | 5.337333 | 0.006 | -25.01477 | -4.092806 |
| -127 | -11.38517 | 5.337333 | 0.033 | -21.84615 | -.9241849 |
| **eTable 3 (continued)** | | | | | |
| -126 | -8.915 | 5.337333 | 0.095 | -19.37598 | 1.545981 |
| -125 | -8.528272 | 5.337333 | 0.110 | -18.98925 | 1.932708 |
| -124 | -10.03264 | 5.108856 | 0.050 | -20.04581 | -.0194645 |
| -123 | -9.373537 | 5.108856 | 0.067 | -19.38671 | .6396362 |
| -122 | -11.23343 | 5.108856 | 0.028 | -21.2466 | -1.220255 |
| -121 | -12.74386 | 5.108856 | 0.013 | -22.75703 | -2.730684 |
| -120 | -11.42096 | 5.108856 | 0.025 | -21.43413 | -1.407789 |
| -119 | -14.34939 | 5.108856 | 0.005 | -24.36256 | -4.336215 |
| -118 | -14.11828 | 5.108856 | 0.006 | -24.13145 | -4.105105 |
| -117 | -10.10629 | 5.108856 | 0.048 | -20.11946 | -.0931145 |
| -116 | -8.270194 | 5.108856 | 0.105 | -18.28337 | 1.742979 |
| -115 | -9.752161 | 4.923705 | 0.048 | -19.40245 | -.1018752 |
| -114 | -10.86005 | 4.923705 | 0.027 | -20.51033 | -1.209764 |
| -113 | -9.698917 | 4.923705 | 0.049 | -19.3492 | -.048632 |
| -112 | -7.710291 | 4.770373 | 0.106 | -17.06005 | 1.639468 |
| -111 | -9.791983 | 4.921761 | 0.047 | -19.43846 | -.1455086 |
| -110 | -8.175382 | 4.770373 | 0.087 | -17.52514 | 1.174376 |
| -109 | -8.281515 | 4.770373 | 0.083 | -17.63127 | 1.068244 |
| -108 | -6.318784 | 4.770373 | 0.185 | -15.66854 | 3.030975 |
| -107 | -4.905847 | 4.770373 | 0.304 | -14.25561 | 4.443912 |
| -106 | -5.123836 | 4.770373 | 0.283 | -14.47359 | 4.225923 |
| -105 | -5.085604 | 4.770373 | 0.286 | -14.43536 | 4.264155 |
| -104 | -5.983343 | 4.641038 | 0.197 | -15.07961 | 3.112925 |
| -103 | -7.49075 | 4.641038 | 0.107 | -16.58702 | 1.605518 |
| -102 | -6.730783 | 4.641038 | 0.147 | -15.82705 | 2.365484 |
| -101 | -5.753785 | 4.641038 | 0.215 | -14.85005 | 3.342482 |
| -100 | -7.02918 | 4.641038 | 0.130 | -16.12545 | 2.067087 |
| -99 | -5.422595 | 4.641038 | 0.243 | -14.51886 | 3.673672 |
| -98 | -6.709929 | 4.641038 | 0.148 | -15.8062 | 2.386338 |
| -97 | -6.361565 | 4.640562 | 0.170 | -15.4569 | 2.73377 |
| -96 | -7.745719 | 4.640562 | 0.095 | -16.84105 | 1.349616 |
| -95 | -5.177378 | 4.640562 | 0.265 | -14.27271 | 3.917957 |
| -94 | -3.262237 | 4.640562 | 0.482 | -12.35757 | 5.833098 |
| -93 | -3.957219 | 4.640562 | 0.394 | -13.05255 | 5.138116 |
| -92 | -8.2069 | 4.640562 | 0.077 | -17.30223 | .8884354 |
| -91 | -7.721954 | 4.434521 | 0.082 | -16.41346 | .9695469 |
| -90 | -6.980406 | 4.5299 | 0.123 | -15.85885 | 1.898035 |
| -89 | -6.674295 | 4.5299 | 0.141 | -15.55274 | 2.204146 |
| -88 | -5.661971 | 4.5299 | 0.211 | -14.54041 | 3.21647 |
| -87 | -5.267289 | 4.5299 | 0.245 | -14.14573 | 3.611152 |
| -86 | -5.742087 | 4.5299 | 0.205 | -14.62053 | 3.136354 |
| -85 | -5.286857 | 4.434028 | 0.233 | -13.97739 | 3.403677 |
| -84 | -5.233423 | 4.434028 | 0.238 | -13.92396 | 3.457111 |
| -83 | -7.329358 | 4.350606 | 0.092 | -15.85639 | 1.197674 |
| -82 | -5.566094 | 4.350606 | 0.201 | -14.09313 | 2.960937 |
| -81 | -6.497959 | 4.350606 | 0.135 | -15.02499 | 2.029073 |
| -80 | -2.879806 | 4.350606 | 0.508 | -11.40684 | 5.647225 |
| -79 | -3.603156 | 4.350606 | 0.408 | -12.13019 | 4.923876 |
| -78 | -3.118523 | 4.350606 | 0.473 | -11.64555 | 5.408509 |
| -77 | -3.95506 | 4.432897 | 0.372 | -12.64338 | 4.733258 |
| -76 | -3.349862 | 4.276438 | 0.433 | -11.73153 | 5.031802 |
| -75 | -1.756667 | 4.348994 | 0.686 | -10.28054 | 6.767204 |
| -74 | -2.82987 | 4.348994 | 0.515 | -11.35374 | 5.694002 |
| -73 | .3125113 | 4.348994 | 0.943 | -8.21136 | 8.836383 |
| -72 | *Reference* | | | | |
| -71 | -.6907289 | 4.276438 | 0.872 | -9.072392 | 7.690935 |
| -70 | -1.381536 | 4.21237 | 0.743 | -9.637629 | 6.874557 |
| -69 | -1.107943 | 4.21237 | 0.793 | -9.364036 | 7.14815 |
| -68 | .0220818 | 4.21237 | 0.996 | -8.234011 | 8.278175 |
| -67 | -.1904587 | 4.21237 | 0.964 | -8.446552 | 8.065634 |
| **eTable 3 (continued)** | | | | | |
| -66 | -.3428716 | 4.21237 | 0.935 | -8.598964 | 7.913221 |
| -65 | .1908145 | 4.21237 | 0.964 | -8.065278 | 8.446907 |
| -64 | 1.338701 | 4.21237 | 0.751 | -6.917392 | 9.594794 |
| -63 | -.9857005 | 4.276438 | 0.818 | -9.367364 | 7.395963 |
| -62 | -.2204648 | 4.21237 | 0.958 | -8.476558 | 8.035628 |
| -61 | -.808984 | 4.21237 | 0.848 | -9.065077 | 7.447109 |
| -60 | 1.776842 | 4.21237 | 0.673 | -6.47925 | 10.03294 |
| -59 | .6113007 | 4.278221 | 0.886 | -7.773857 | 8.996459 |
| -58 | -1.115747 | 4.278221 | 0.794 | -9.500905 | 7.269411 |
| -57 | -1.813976 | 4.278221 | 0.672 | -10.19913 | 6.571182 |
| -56 | -2.825644 | 4.214292 | 0.503 | -11.08551 | 5.434217 |
| -55 | -2.72554 | 4.155205 | 0.512 | -10.86959 | 5.418513 |
| -54 | -1.109502 | 4.155205 | 0.789 | -9.253555 | 7.034551 |
| -53 | -1.882975 | 4.155205 | 0.650 | -10.02703 | 6.261078 |
| -52 | -.9885107 | 4.155205 | 0.812 | -9.132564 | 7.155542 |
| -51 | -.85991 | 4.155205 | 0.836 | -9.003963 | 7.284143 |
| -50 | -.7871512 | 4.103854 | 0.848 | -8.830558 | 7.256256 |
| -49 | -1.536584 | 4.103854 | 0.708 | -9.579991 | 6.506823 |
| -48 | -1.588011 | 4.103854 | 0.699 | -9.631418 | 6.455396 |
| -47 | -2.034448 | 4.103854 | 0.620 | -10.07785 | 6.008959 |
| -46 | -1.472658 | 4.103854 | 0.720 | -9.516065 | 6.570749 |
| -45 | .6227991 | 4.057511 | 0.878 | -7.329776 | 8.575374 |
| -44 | -.7267829 | 4.015313 | 0.856 | -8.596652 | 7.143086 |
| -43 | .1378463 | 4.015313 | 0.973 | -7.732023 | 8.007715 |
| -42 | -.8375212 | 4.058515 | 0.837 | -8.792065 | 7.117023 |
| -41 | .0379382 | 4.058515 | 0.993 | -7.916606 | 7.992482 |
| -40 | 1.430308 | 4.015313 | 0.722 | -6.439562 | 9.300177 |
| -39 | .8478307 | 4.015313 | 0.833 | -7.022039 | 8.7177 |
| -38 | .8129184 | 4.015313 | 0.840 | -7.056951 | 8.682788 |
| -37 | .6768081 | 4.015313 | 0.866 | -7.193061 | 8.546677 |
| -36 | .9414724 | 4.058515 | 0.817 | -7.013072 | 8.896016 |
| -35 | 2.302022 | 3.977014 | 0.563 | -5.492781 | 10.09683 |
| -34 | 3.404451 | 4.0166 | 0.397 | -4.467941 | 11.27684 |
| -33 | 4.264136 | 4.0166 | 0.288 | -3.608256 | 12.13653 |
| -32 | 4.643559 | 4.0166 | 0.248 | -3.228832 | 12.51595 |
| -31 | 6.94144 | 3.941924 | 0.078 | -.7845896 | 14.66747 |
| -30 | 6.799914 | 3.941924 | 0.085 | -.9261149 | 14.52594 |
| -29 | 6.95795 | 3.941924 | 0.078 | -.7680793 | 14.68398 |
| -28 | 7.316006 | 3.941924 | 0.063 | -.410023 | 15.04204 |
| -27 | 8.865572 | 3.941924 | 0.025 | 1.139543 | 16.5916 |
| -26 | 7.853043 | 3.941924 | 0.046 | .1270134 | 15.57907 |
| -25 | 6.501389 | 3.941924 | 0.099 | -1.22464 | 14.22742 |
| -24 | 7.070778 | 3.941924 | 0.073 | -.655251 | 14.79681 |
| -23 | 7.657436 | 3.941924 | 0.052 | -.0685929 | 15.38347 |
| -22 | 7.836156 | 3.941924 | 0.047 | .1101266 | 15.56218 |
| -21 | 7.52613 | 3.910079 | 0.054 | -.1374834 | 15.18974 |
| -20 | 7.926334 | 3.880873 | 0.041 | .3199622 | 15.53271 |
| -19 | 8.48885 | 3.912811 | 0.030 | .8198809 | 16.15782 |
| -18 | 8.994986 | 3.912811 | 0.022 | 1.326016 | 16.66396 |
| -17 | 9.695761 | 3.854452 | 0.012 | 2.141175 | 17.25035 |
| -16 | 8.477568 | 3.829389 | 0.027 | .972103 | 15.98303 |
| -15 | 9.909553 | 3.829389 | 0.010 | 2.404087 | 17.41502 |
| -14 | 10.50892 | 3.829389 | 0.006 | 3.003454 | 18.01439 |
| -13 | 12.41479 | 3.829389 | 0.001 | 4.909325 | 19.92026 |
| -12 | 13.46584 | 3.806454 | 0.000 | 6.005328 | 20.92635 |
| -11 | 13.80154 | 3.785189 | 0.000 | 6.382706 | 21.22037 |
| -10 | 13.29287 | 3.785189 | 0.000 | 5.874033 | 20.7117 |
| -9 | 13.87682 | 3.785189 | 0.000 | 6.457987 | 21.29565 |
| -8 | 14.66132 | 3.785189 | 0.000 | 7.24249 | 22.08016 |
| -7 | 13.66834 | 3.80829 | 0.000 | 6.204226 | 21.13245 |
| **eTable 3 (continued)** | | | | | |
| -6 | 11.85817 | 3.80829 | 0.002 | 4.394053 | 19.32228 |
| -5 | 9.048849 | 3.785189 | 0.017 | 1.630016 | 16.46768 |
| -4 | 8.770952 | 3.806676 | 0.021 | 1.310005 | 16.2319 |
| -3 | 6.947222 | 3.806676 | 0.068 | -.5137249 | 14.40817 |
| -2 | 1.797559 | 3.832957 | 0.639 | -5.714899 | 9.310016 |
| -1 | -17.89006 | 3.918814 | 0.000 | -25.57079 | -10.20932 |
| Hour of death | -28.44833 | 4.121847 | 0.000 | -36.527 | -20.36966 |

**eTable 4** The association between time before death in hours and respiratory rate in breaths per minute.

Output following linear mixed effects model with hour 72 before death as the reference.

| **Hour** | **Coefficient** | **Standard Error** | **p-value** | **95 % CI (lower)** | **95% CI (upper)** |
| --- | --- | --- | --- | --- | --- |
| -182 | -5.517409 | 3.435741 | 0.108 | -12.25134 | 1.21652 |
| -181 | -.8818772 | 3.435741 | 0.797 | -7.615807 | 5.852052 |
| -180 | -3.969341 | 3.435741 | 0.248 | -10.70327 | 2.764589 |
| -179 | -3.141128 | 2.504035 | 0.210 | -8.048946 | 1.76669 |
| -178 | -3.151122 | 2.504035 | 0.208 | -8.05894 | 1.756696 |
| -177 | -1.887357 | 2.504035 | 0.451 | -6.795175 | 3.020461 |
| -176 | -1.265022 | 2.504035 | 0.613 | -6.17284 | 3.642796 |
| -175 | -1.001753 | 2.504035 | 0.689 | -5.909571 | 3.906065 |
| -174 | 1.317656 | 2.504035 | 0.599 | -3.590162 | 6.225473 |
| -173 | 1.341156 | 2.504035 | 0.592 | -3.566662 | 6.248974 |
| -172 | .9487611 | 2.504035 | 0.705 | -3.959057 | 5.856579 |
| -171 | .7053772 | 2.504035 | 0.778 | -4.202441 | 5.613195 |
| -170 | -1.059594 | 2.504035 | 0.672 | -5.967412 | 3.848224 |
| -169 | -1.861008 | 2.504035 | 0.457 | -6.768825 | 3.04681 |
| -168 | -.7511648 | 2.103607 | 0.721 | -4.874158 | 3.371828 |
| -167 | -.2059788 | 2.103607 | 0.922 | -4.328972 | 3.917014 |
| -166 | -.2373229 | 2.103607 | 0.910 | -4.360316 | 3.88567 |
| -165 | .6971261 | 2.103607 | 0.740 | -3.425867 | 4.820119 |
| -164 | -.6376167 | 2.103607 | 0.762 | -4.76061 | 3.485376 |
| -163 | -.8629604 | 2.103607 | 0.682 | -4.985954 | 3.260033 |
| -162 | .6471652 | 2.103607 | 0.758 | -3.475828 | 4.770158 |
| -161 | -1.233042 | 2.103607 | 0.558 | -5.356035 | 2.889951 |
| -160 | -.324355 | 1.871301 | 0.862 | -3.992037 | 3.343327 |
| -159 | -.6093466 | 1.871301 | 0.745 | -4.277029 | 3.058336 |
| -158 | .2885316 | 1.871301 | 0.877 | -3.379151 | 3.956214 |
| -157 | -.3044199 | 1.871301 | 0.871 | -3.972102 | 3.363262 |
| -156 | -1.780939 | 1.71689 | 0.300 | -5.145981 | 1.584103 |
| -155 | -1.729931 | 1.71689 | 0.314 | -5.094973 | 1.635111 |
| -154 | .1461915 | 1.71689 | 0.932 | -3.21885 | 3.511233 |
| -153 | -.9918401 | 1.605717 | 0.537 | -4.138987 | 2.155307 |
| -152 | -1.628996 | 1.605717 | 0.310 | -4.776143 | 1.518151 |
| -151 | -1.473069 | 1.605717 | 0.359 | -4.620216 | 1.674077 |
| -150 | -1.954811 | 1.716899 | 0.255 | -5.319872 | 1.41025 |
| -149 | -1.678369 | 1.716899 | 0.328 | -5.04343 | 1.686692 |
| -148 | -1.588114 | 1.716899 | 0.355 | -4.953174 | 1.776947 |
| -147 | -1.288082 | 1.716899 | 0.453 | -4.653142 | 2.076979 |
| -146 | -1.805653 | 1.716899 | 0.293 | -5.170714 | 1.559408 |
| -145 | -.7250039 | 1.716899 | 0.673 | -4.090065 | 2.640057 |
| -144 | -.6653178 | 1.716899 | 0.698 | -4.030379 | 2.699743 |
| -143 | -.2390507 | 1.716899 | 0.889 | -3.604112 | 3.12601 |
| -142 | -1.455765 | 1.716899 | 0.396 | -4.820825 | 1.909296 |
| -141 | -2.014452 | 1.716899 | 0.241 | -5.379513 | 1.350608 |
| -140 | -1.861154 | 1.521295 | 0.221 | -4.842838 | 1.12053 |
| -139 | -2.688733 | 1.521295 | 0.077 | -5.670417 | .2929514 |
| -138 | -3.050227 | 1.521295 | 0.045 | -6.031911 | -.0685424 |
| -137 | -3.195035 | 1.521295 | 0.036 | -6.176719 | -.2133504 |
| -136 | -3.472028 | 1.521295 | 0.022 | -6.453712 | -.4903437 |
| -135 | -3.066473 | 1.521295 | 0.044 | -6.048157 | -.0847892 |
| -134 | -2.637893 | 1.521295 | 0.083 | -5.619577 | .3437915 |
| -133 | -2.227111 | 1.521295 | 0.143 | -5.208795 | .7545736 |
| -132 | -2.537406 | 1.521295 | 0.095 | -5.51909 | .4442782 |
| **eTable 4 (continued)** | | | | | |
| -131 | -2.267711 | 1.521295 | 0.136 | -5.249395 | .7139731 |
| -130 | -1.918456 | 1.521295 | 0.207 | -4.90014 | 1.063228 |
| -129 | -1.69123 | 1.521295 | 0.266 | -4.672915 | 1.290454 |
| -128 | -1.493667 | 1.521295 | 0.326 | -4.475352 | 1.488017 |
| -127 | -2.544624 | 1.521295 | 0.094 | -5.526308 | .4370598 |
| -126 | -2.231082 | 1.521295 | 0.142 | -5.212766 | .7506025 |
| -125 | -1.097155 | 1.521295 | 0.471 | -4.078839 | 1.884529 |
| -124 | .1280863 | 1.454709 | 0.930 | -2.723091 | 2.979264 |
| -123 | -.1039502 | 1.454709 | 0.943 | -2.955128 | 2.747227 |
| -122 | -.2050861 | 1.454709 | 0.888 | -3.056264 | 2.646091 |
| -121 | -.6660143 | 1.454709 | 0.647 | -3.517192 | 2.185163 |
| -120 | -.7824303 | 1.454709 | 0.591 | -3.633608 | 2.068747 |
| -119 | -1.853846 | 1.454709 | 0.203 | -4.705023 | .9973318 |
| -118 | -1.947868 | 1.454709 | 0.181 | -4.799046 | .9033091 |
| -117 | -.7726096 | 1.454709 | 0.595 | -3.623787 | 2.078568 |
| -116 | -.2171224 | 1.454709 | 0.881 | -3.0683 | 2.634055 |
| -115 | -1.45646 | 1.400702 | 0.298 | -4.201785 | 1.288865 |
| -114 | -1.981398 | 1.400702 | 0.157 | -4.726723 | .7639275 |
| -113 | -1.167097 | 1.400702 | 0.405 | -3.912422 | 1.578228 |
| -112 | -.9020155 | 1.355939 | 0.506 | -3.559606 | 1.755575 |
| -111 | -.5507033 | 1.355939 | 0.685 | -3.208294 | 2.106888 |
| -110 | -1.190096 | 1.355939 | 0.380 | -3.847687 | 1.467495 |
| -109 | -1.806188 | 1.355939 | 0.183 | -4.463779 | .8514024 |
| -108 | -1.560583 | 1.355939 | 0.250 | -4.218174 | 1.097008 |
| -107 | .2597192 | 1.355939 | 0.848 | -2.397872 | 2.91731 |
| -106 | .0586086 | 1.355939 | 0.966 | -2.598982 | 2.716199 |
| -105 | -.5934093 | 1.355939 | 0.662 | -3.251 | 2.064182 |
| -104 | -1.893394 | 1.31816 | 0.151 | -4.47694 | .6901517 |
| -103 | -1.940423 | 1.31816 | 0.141 | -4.523969 | .6431228 |
| -102 | -.5164836 | 1.31816 | 0.695 | -3.100029 | 2.067062 |
| -101 | -.629022 | 1.31816 | 0.633 | -3.212568 | 1.954524 |
| -100 | -1.315457 | 1.31816 | 0.318 | -3.899003 | 1.268089 |
| -99 | -.6106391 | 1.31816 | 0.643 | -3.194185 | 1.972907 |
| -98 | -.3290481 | 1.31816 | 0.803 | -2.912594 | 2.254498 |
| -97 | -.2875912 | 1.318049 | 0.827 | -2.870919 | 2.295737 |
| -96 | -1.169799 | 1.318049 | 0.375 | -3.753127 | 1.41353 |
| -95 | -.3828544 | 1.318049 | 0.771 | -2.966183 | 2.200474 |
| -94 | -.5892513 | 1.318049 | 0.655 | -3.17258 | 1.994077 |
| -93 | -.9843922 | 1.318049 | 0.455 | -3.56772 | 1.598936 |
| -92 | -.7248391 | 1.318049 | 0.582 | -3.308167 | 1.858489 |
| -91 | -1.490104 | 1.257768 | 0.236 | -3.955284 | .9750752 |
| -90 | -2.300594 | 1.285698 | 0.074 | -4.820516 | .2193274 |
| -89 | -2.645202 | 1.285698 | 0.040 | -5.165123 | -.1252801 |
| -88 | -1.268069 | 1.285698 | 0.324 | -3.787991 | 1.251852 |
| -87 | -1.784285 | 1.285698 | 0.165 | -4.304207 | .7356362 |
| -86 | -2.265858 | 1.285698 | 0.078 | -4.78578 | .2540634 |
| -85 | -1.837069 | 1.257648 | 0.144 | -4.302015 | .6278763 |
| -84 | -1.388229 | 1.257648 | 0.270 | -3.853174 | 1.076717 |
| -83 | -2.187006 | 1.233201 | 0.076 | -4.604036 | .2300247 |
| -82 | -1.77242 | 1.233201 | 0.151 | -4.18945 | .6446104 |
| -81 | -1.728163 | 1.233201 | 0.161 | -4.145193 | .6888674 |
| -80 | -1.390246 | 1.233201 | 0.260 | -3.807276 | 1.026784 |
| -79 | -2.254533 | 1.233201 | 0.068 | -4.671563 | .162497 |
| -78 | -.7862147 | 1.233201 | 0.524 | -3.203245 | 1.630816 |
| **eTable 4 (continued)** | | | | | |
| -77 | -.593149 | 1.233201 | 0.631 | -3.010179 | 1.823881 |
| -76 | -.556547 | 1.211483 | 0.646 | -2.931011 | 1.817917 |
| -75 | -.1854602 | 1.211483 | 0.878 | -2.559924 | 2.189003 |
| -74 | .0527176 | 1.211483 | 0.965 | -2.321746 | 2.427181 |
| -73 | .349462 | 1.211483 | 0.773 | -2.025002 | 2.723926 |
| -72 | *Reference* |  |  |  |  |
| -71 | -.8727419 | 1.211483 | 0.471 | -3.247206 | 1.501722 |
| -70 | -1.48861 | 1.194673 | 0.213 | -3.830126 | .8529057 |
| -69 | -1.630871 | 1.194673 | 0.172 | -3.972387 | .7106451 |
| -68 | -1.367474 | 1.194673 | 0.252 | -3.70899 | .974042 |
| -67 | -1.531439 | 1.194673 | 0.200 | -3.872955 | .8100768 |
| -66 | -2.219076 | 1.194673 | 0.063 | -4.560593 | .1224396 |
| -65 | -1.696795 | 1.194673 | 0.156 | -4.038311 | .6447212 |
| -64 | -1.640472 | 1.194673 | 0.170 | -3.981988 | .7010437 |
| -63 | -1.072859 | 1.194673 | 0.369 | -3.414375 | 1.268657 |
| -62 | -1.102815 | 1.211483 | 0.363 | -3.477278 | 1.271649 |
| -61 | -1.261302 | 1.211483 | 0.298 | -3.635765 | 1.113162 |
| -60 | -1.491631 | 1.211483 | 0.218 | -3.866094 | .8828331 |
| -59 | -1.481182 | 1.211483 | 0.221 | -3.855646 | .8932816 |
| -58 | -1.753061 | 1.211483 | 0.148 | -4.127525 | .6214024 |
| -57 | -1.941802 | 1.211483 | 0.109 | -4.316266 | .4326615 |
| -56 | -1.332512 | 1.17595 | 0.257 | -3.637332 | .9723082 |
| -55 | -2.156887 | 1.17595 | 0.067 | -4.461707 | .1479335 |
| -54 | -2.050537 | 1.17595 | 0.081 | -4.355357 | .2542835 |
| -53 | -2.058601 | 1.17595 | 0.080 | -4.363421 | .2462188 |
| -52 | -1.744345 | 1.17595 | 0.138 | -4.049165 | .5604752 |
| -51 | -2.163629 | 1.17595 | 0.066 | -4.468449 | .141191 |
| -50 | -2.342144 | 1.17595 | 0.046 | -4.646964 | -.0373237 |
| -49 | -2.408576 | 1.17595 | 0.041 | -4.713396 | -.1037562 |
| -48 | -2.336745 | 1.17595 | 0.047 | -4.641566 | -.0319254 |
| -47 | -1.667724 | 1.17595 | 0.156 | -3.972544 | .6370958 |
| -46 | -1.567465 | 1.17595 | 0.183 | -3.872285 | .7373548 |
| -45 | -.8627893 | 1.160896 | 0.457 | -3.138104 | 1.412525 |
| -44 | -1.09971 | 1.160896 | 0.343 | -3.375024 | 1.175605 |
| -43 | -1.92674 | 1.147272 | 0.093 | -4.175351 | .3218713 |
| -42 | -1.858416 | 1.147272 | 0.105 | -4.107027 | .3901953 |
| -41 | -1.895117 | 1.147272 | 0.099 | -4.143728 | .3534937 |
| -40 | -2.81998 | 1.147272 | 0.014 | -5.068591 | -.5713695 |
| -39 | -2.999973 | 1.147272 | 0.009 | -5.248584 | -.7513619 |
| -38 | -3.213121 | 1.147272 | 0.005 | -5.461732 | -.9645103 |
| -37 | -3.34837 | 1.147272 | 0.004 | -5.596981 | -1.099759 |
| -36 | -2.226655 | 1.147272 | 0.052 | -4.475266 | .021956 |
| -35 | -1.835316 | 1.135171 | 0.106 | -4.060211 | .3895789 |
| -34 | -2.262707 | 1.135171 | 0.046 | -4.487602 | -.0378119 |
| -33 | -1.477803 | 1.135171 | 0.193 | -3.702698 | .7470919 |
| -32 | -.6898619 | 1.135171 | 0.543 | -2.914757 | 1.535033 |
| -31 | -.5353662 | 1.123899 | 0.634 | -2.738168 | 1.667436 |
| -30 | -.2283357 | 1.123899 | 0.839 | -2.431138 | 1.974467 |
| -29 | .4398145 | 1.123899 | 0.696 | -1.762988 | 2.642617 |
| -28 | .3973634 | 1.123899 | 0.724 | -1.805439 | 2.600166 |
| -27 | .0211625 | 1.123899 | 0.985 | -2.18164 | 2.223965 |
| -26 | .1126235 | 1.134967 | 0.921 | -2.111871 | 2.337118 |
| -25 | .3912166 | 1.134967 | 0.730 | -1.833278 | 2.615711 |
| -24 | -.2138986 | 1.134967 | 0.851 | -2.438393 | 2.010596 |
| **eTable 4 (continued)** | | | | | |
| -23 | -.4370681 | 1.134967 | 0.700 | -2.661563 | 1.787427 |
| -22 | -.3474929 | 1.134967 | 0.759 | -2.571988 | 1.877002 |
| -21 | -.2181478 | 1.123882 | 0.846 | -2.420916 | 1.98462 |
| -20 | -.0099202 | 1.113753 | 0.993 | -2.192835 | 2.172995 |
| -19 | -.5228142 | 1.113753 | 0.639 | -2.705729 | 1.660101 |
| -18 | -.8366925 | 1.113753 | 0.453 | -3.019607 | 1.346223 |
| -17 | -.355153 | 1.104575 | 0.748 | -2.520079 | 1.809773 |
| -16 | .0266826 | 1.096016 | 0.981 | -2.12147 | 2.174835 |
| -15 | .1125381 | 1.096016 | 0.918 | -2.035614 | 2.260691 |
| -14 | -.2619007 | 1.096016 | 0.811 | -2.410053 | 1.886252 |
| -13 | -.2535028 | 1.08793 | 0.816 | -2.385806 | 1.878801 |
| -12 | -.072245 | 1.080686 | 0.947 | -2.19035 | 2.04586 |
| -11 | .37246 | 1.080686 | 0.730 | -1.745645 | 2.490565 |
| -10 | .6123375 | 1.074035 | 0.569 | -1.492733 | 2.717408 |
| -9 | .4467353 | 1.074035 | 0.677 | -1.658335 | 2.551806 |
| -8 | .5013605 | 1.074035 | 0.641 | -1.60371 | 2.606431 |
| -7 | .8055235 | 1.074035 | 0.453 | -1.299547 | 2.910594 |
| -6 | .9372664 | 1.074035 | 0.383 | -1.167804 | 3.042337 |
| -5 | .9034398 | 1.074035 | 0.400 | -1.201631 | 3.00851 |
| -4 | .4608831 | 1.074035 | 0.668 | -1.644187 | 2.565953 |
| -3 | .0931599 | 1.074035 | 0.931 | -2.01191 | 2.19823 |
| -2 | -1.237875 | 1.080892 | 0.252 | -3.356384 | .8806349 |
| -1 | -4.224652 | 1.106913 | 0.000 | -6.394162 | -2.055142 |
| Hour of death | -6.647593 | 1.151859 | 0.000 | -8.905195 | -4.389991 |

**eTable 5** The association between time before death in hours and body temperature in ° Celsius.

Output following linear mixed effects model with hour 72 before death as the reference.

| **Hour** | **Coefficient** | **Standard Error** | **p-value** | **95 % CI (lower)** | **95% CI (upper)** |
| --- | --- | --- | --- | --- | --- |
| -192 | -.0784119 | .3802166 | 0.837 | -.8236227 | .6667989 |
| -191 | .3225848 | .2762045 | 0.243 | -.218766 | .8639356 |
| -190 | .4300554 | .2762045 | 0.119 | -.1112954 | .9714062 |
| -189 | .3461173 | .2762045 | 0.210 | -.1952336 | .8874681 |
| -188 | .2565968 | .2762045 | 0.353 | -.284754 | .7979476 |
| -187 | .2787956 | .2762045 | 0.313 | -.2625552 | .8201465 |
| -186 | .1917741 | .2762045 | 0.487 | -.3495767 | .7331249 |
| -185 | .0967683 | .2762045 | 0.726 | -.4445825 | .6381191 |
| -184 | .0907884 | .2762045 | 0.742 | -.4505624 | .6321392 |
| -183 | .0567389 | .2762045 | 0.837 | -.484612 | .5980897 |
| -182 | -.0976596 | .2313524 | 0.673 | -.551102 | .3557828 |
| -181 | -.0981335 | .2313524 | 0.671 | -.5515759 | .3553089 |
| -180 | -.0061099 | .2313524 | 0.979 | -.4595523 | .4473324 |
| -179 | -.0615285 | .2313524 | 0.790 | -.5149709 | .3919138 |
| -178 | -.0675985 | .2052809 | 0.742 | -.4699417 | .3347447 |
| -177 | -.0697832 | .2052809 | 0.734 | -.4721264 | .33256 |
| -176 | -.0140386 | .2052809 | 0.945 | -.4163818 | .3883046 |
| -175 | .0780063 | .2052809 | 0.704 | -.3243369 | .4803495 |
| -174 | .1251579 | .2052809 | 0.542 | -.2771853 | .527501 |
| -173 | .0808658 | .2052809 | 0.694 | -.3214774 | .483209 |
| -172 | .1144183 | .2052809 | 0.577 | -.2879249 | .5167614 |
| -171 | .1323367 | .2052809 | 0.519 | -.2700065 | .5346799 |
| -170 | .14384 | .2052809 | 0.483 | -.2585032 | .5461832 |
| -169 | .124522 | .2052809 | 0.544 | -.2778212 | .5268651 |
| -168 | .1529015 | .2052809 | 0.456 | -.2494417 | .5552447 |
| -167 | .1520839 | .2052809 | 0.459 | -.2502592 | .5544271 |
| -166 | .0783345 | .1879073 | 0.677 | -.2899569 | .446626 |
| -165 | .0048849 | .1879073 | 0.979 | -.3634066 | .3731763 |
| -164 | .0928864 | .1879073 | 0.621 | -.2754051 | .4611779 |
| -163 | .2182834 | .1879073 | 0.245 | -.1500081 | .5865748 |
| -162 | .2334557 | .1879073 | 0.214 | -.1348358 | .6017471 |
| -161 | .1026682 | .1879073 | 0.585 | -.2656232 | .4709597 |
| -160 | -.032565 | .1753604 | 0.853 | -.3762651 | .3111351 |
| -159 | -.0491482 | .1753604 | 0.779 | -.3928483 | .2945519 |
| -158 | -.0149328 | .1753604 | 0.932 | -.3586329 | .3287673 |
| -157 | -.0340888 | .1753604 | 0.846 | -.3777889 | .3096113 |
| -156 | -.1401981 | .1753604 | 0.424 | -.4838982 | .203502 |
| -155 | -.1540817 | .1658182 | 0.353 | -.4790794 | .170916 |
| -154 | -.1834053 | .1658182 | 0.269 | -.5084031 | .1415924 |
| -153 | -.1851892 | .1658182 | 0.264 | -.5101869 | .1398085 |
| -152 | -.0957786 | .1582839 | 0.545 | -.4060093 | .214452 |
| -151 | -.0645948 | .1582839 | 0.683 | -.3748255 | .2456359 |
| -150 | .0704353 | .1658189 | 0.671 | -.2545637 | .3954343 |
| -149 | .068139 | .1658189 | 0.681 | -.25686 | .393138 |
| -148 | .0531583 | .1658189 | 0.749 | -.2718407 | .3781572 |
| -147 | -.0194141 | .1658189 | 0.907 | -.3444131 | .3055849 |
| -146 | .0054219 | .1658189 | 0.974 | -.3195771 | .3304209 |
| -145 | -.0455307 | .1658189 | 0.784 | -.3705297 | .2794682 |
| -144 | -.0087283 | .1658189 | 0.958 | -.3337273 | .3162707 |
| -143 | .0106203 | .1658189 | 0.949 | -.3143786 | .3356193 |
| -142 | -.0093375 | .1658189 | 0.955 | -.3343365 | .3156615 |
| **eTable 5 (continued)** | | | | | |
| -141 | -.0181938 | .1658189 | 0.913 | -.3431928 | .3068052 |
| -140 | .0313338 | .1582839 | 0.843 | -.2788969 | .3415645 |
| -139 | -.0135075 | .1582839 | 0.932 | -.3237382 | .2967231 |
| -138 | -.1073275 | .1582839 | 0.498 | -.4175581 | .2029032 |
| -137 | -.1669262 | .1521619 | 0.273 | -.465158 | .1313056 |
| -136 | -.0796294 | .1521619 | 0.601 | -.3778612 | .2186024 |
| -135 | -.0662517 | .1521619 | 0.663 | -.3644835 | .2319801 |
| -134 | -.0956115 | .1521619 | 0.530 | -.3938433 | .2026202 |
| -133 | -.0773624 | .1521619 | 0.611 | -.3755942 | .2208693 |
| -132 | -.1139004 | .1521619 | 0.454 | -.4121322 | .1843314 |
| -131 | -.0748475 | .1521619 | 0.623 | -.3730793 | .2233843 |
| -130 | -.0959761 | .1521619 | 0.528 | -.3942079 | .2022557 |
| -129 | -.01082 | .1521619 | 0.943 | -.3090518 | .2874118 |
| -128 | -.0566728 | .1521619 | 0.710 | -.3549046 | .241559 |
| -127 | -.0734223 | .1521619 | 0.629 | -.3716541 | .2248095 |
| -126 | -.0514758 | .1521619 | 0.735 | -.3497076 | .246756 |
| -125 | -.0209217 | .1521619 | 0.891 | -.3191535 | .27731 |
| -124 | -.1085496 | .1521619 | 0.476 | -.4067813 | .1896822 |
| -123 | -.1793328 | .1470768 | 0.223 | -.4675981 | .1089325 |
| -122 | -.1960435 | .1470768 | 0.183 | -.4843088 | .0922217 |
| -121 | -.2197795 | .1470768 | 0.135 | -.5080448 | .0684858 |
| -120 | -.2413215 | .1470768 | 0.101 | -.5295868 | .0469438 |
| -119 | -.2366394 | .1470768 | 0.108 | -.5249047 | .0516259 |
| -118 | -.3343168 | .1470768 | 0.023 | -.6225821 | -.0460515 |
| -117 | -.3020998 | .1470768 | 0.040 | -.5903651 | -.0138345 |
| -116 | -.2457435 | .1470768 | 0.095 | -.5340087 | .0425218 |
| -115 | -.2596555 | .1470768 | 0.077 | -.5479208 | .0286098 |
| -114 | -.2155703 | .1427791 | 0.131 | -.4954122 | .0642715 |
| -113 | -.167401 | .1427791 | 0.241 | -.4472428 | .1124408 |
| -112 | -.1976935 | .1427791 | 0.166 | -.4775353 | .0821483 |
| -111 | -.1799001 | .1390954 | 0.196 | -.4525221 | .0927218 |
| -110 | -.2120177 | .1390954 | 0.127 | -.4846397 | .0606042 |
| -109 | -.2313361 | .1390954 | 0.096 | -.5039581 | .0412858 |
| -108 | -.2043717 | .1390954 | 0.142 | -.4769936 | .0682503 |
| -107 | -.1793359 | .1390954 | 0.197 | -.4519578 | .0932861 |
| -106 | -.1952194 | .1390954 | 0.160 | -.4678413 | .0774026 |
| -105 | -.1322323 | .1390954 | 0.342 | -.4048542 | .1403897 |
| -104 | -.1004776 | .1358986 | 0.460 | -.366834 | .1658789 |
| -103 | -.1312394 | .1358986 | 0.334 | -.3975959 | .135117 |
| -102 | -.0821089 | .1358986 | 0.546 | -.3484653 | .1842476 |
| -101 | -.0742381 | .1358986 | 0.585 | -.3405945 | .1921183 |
| -100 | -.1219994 | .1358986 | 0.369 | -.3883558 | .1443571 |
| -99 | -.1233819 | .1358986 | 0.364 | -.3897384 | .1429745 |
| -98 | -.1415639 | .1358986 | 0.298 | -.4079204 | .1247925 |
| -97 | -.1388155 | .1390858 | 0.318 | -.4114187 | .1337877 |
| -96 | -.1745919 | .1358886 | 0.199 | -.4409286 | .0917447 |
| -95 | -.140349 | .1358886 | 0.302 | -.4066857 | .1259876 |
| -94 | -.1457753 | .1358886 | 0.283 | -.412112 | .1205613 |
| -93 | -.2058421 | .1358886 | 0.130 | -.4721787 | .0604946 |
| -92 | -.2673334 | .1358886 | 0.049 | -.5336701 | -.0009967 |
| -91 | -.2936372 | .1330953 | 0.027 | -.5544992 | -.0327751 |
| -90 | -.2587259 | .133085 | 0.052 | -.5195677 | .0021159 |
| -89 | -.2236828 | .133085 | 0.093 | -.4845246 | .037159 |
| -88 | -.2493495 | .133085 | 0.061 | -.5101913 | .0114923 |
| **eTable 5 (continued)** | | | | | |
| -87 | -.2419964 | .133085 | 0.069 | -.5028382 | .0188454 |
| -86 | -.2290157 | .133085 | 0.085 | -.4898575 | .0318261 |
| -85 | -.2458865 | .133085 | 0.065 | -.5067283 | .0149553 |
| -84 | -.2032063 | .133085 | 0.127 | -.4640481 | .0576355 |
| -83 | -.1834039 | .1306155 | 0.160 | -.4394056 | .0725977 |
| -82 | -.1459644 | .1306155 | 0.264 | -.401966 | .1100372 |
| -81 | -.1241605 | .1306155 | 0.342 | -.3801621 | .1318411 |
| -80 | -.0602698 | .1306155 | 0.644 | -.3162715 | .1957318 |
| -79 | -.0082029 | .1284024 | 0.949 | -.259867 | .2434613 |
| -78 | -.0472422 | .1284024 | 0.713 | -.2989064 | .2044219 |
| -77 | -.0623497 | .1284024 | 0.627 | -.3140138 | .1893145 |
| -76 | -.0242094 | .1264172 | 0.848 | -.2719825 | .2235637 |
| -75 | -.0417355 | .1264172 | 0.741 | -.2895086 | .2060375 |
| -74 | -.0225033 | .1264172 | 0.859 | -.2702764 | .2252697 |
| -73 | -.0052103 | .1264172 | 0.967 | -.2529833 | .2425628 |
| -72 | *Reference* |  |  |  |  |
| -71 | -.0448983 | .1264172 | 0.722 | -.2926714 | .2028747 |
| -70 | -.1130322 | .1264172 | 0.371 | -.3608053 | .1347409 |
| -69 | -.1394171 | .1248974 | 0.264 | -.3842115 | .1053772 |
| -68 | -.1252902 | .1248974 | 0.316 | -.3700846 | .1195041 |
| -67 | -.1089129 | .1248974 | 0.383 | -.3537073 | .1358815 |
| -66 | -.1324487 | .1248974 | 0.289 | -.3772431 | .1123456 |
| -65 | -.1518576 | .1248974 | 0.224 | -.396652 | .0929368 |
| -64 | -.0348202 | .1248974 | 0.780 | -.2796146 | .2099741 |
| -63 | -.0026242 | .1248974 | 0.983 | -.2474186 | .2421702 |
| -62 | -.0249172 | .1264172 | 0.844 | -.2726903 | .2228558 |
| -61 | -.0622764 | .1264172 | 0.622 | -.3100495 | .1854967 |
| -60 | -.0416653 | .1264172 | 0.742 | -.2894384 | .2061078 |
| -59 | .0034615 | .1264172 | 0.978 | -.2443116 | .2512346 |
| -58 | .0364605 | .1264172 | 0.773 | -.2113126 | .2842335 |
| -57 | .0165565 | .1264172 | 0.896 | -.2312166 | .2643296 |
| -56 | -.0036339 | .1264172 | 0.977 | -.251407 | .2441391 |
| -55 | -.0162147 | .1246807 | 0.897 | -.2605843 | .228155 |
| -54 | -.0071972 | .1231068 | 0.953 | -.248482 | .2340877 |
| -53 | -.0309013 | .1231068 | 0.802 | -.2721862 | .2103836 |
| -52 | -.0648358 | .1231068 | 0.598 | -.3061207 | .1764491 |
| -51 | -.1037556 | .1231068 | 0.399 | -.3450405 | .1375293 |
| -50 | -.1338423 | .1231068 | 0.277 | -.3751272 | .1074426 |
| -49 | -.1457025 | .1231068 | 0.237 | -.3869874 | .0955823 |
| -48 | -.1915333 | .1231068 | 0.120 | -.4328181 | .0497516 |
| -47 | -.1387995 | .1231068 | 0.260 | -.3800843 | .1024854 |
| -46 | -.0659993 | .1231068 | 0.592 | -.3072841 | .1752856 |
| -45 | -.0873657 | .1231068 | 0.478 | -.3286506 | .1539192 |
| -44 | -.0144281 | .1216788 | 0.906 | -.2529142 | .224058 |
| -43 | -.022416 | .1203729 | 0.852 | -.2583426 | .2135106 |
| -42 | .0371515 | .1203729 | 0.758 | -.1987751 | .2730782 |
| -41 | .060295 | .1203729 | 0.616 | -.1756316 | .2962216 |
| -40 | .0203263 | .1203729 | 0.866 | -.2156004 | .2562529 |
| -39 | -.0139093 | .1203729 | 0.908 | -.249836 | .2220173 |
| -38 | -.0112456 | .1203729 | 0.926 | -.2471722 | .224681 |
| -37 | .0081294 | .1203729 | 0.946 | -.2277972 | .2440561 |
| -36 | .0094479 | .1203729 | 0.937 | -.2264788 | .2453745 |
| -35 | -.0309026 | .1203729 | 0.797 | -.2668292 | .205024 |
| -34 | -.0115028 | .1192012 | 0.923 | -.2451327 | .2221272 |
| **eTable 5 (continued)** | | | | | |
| -33 | .0738496 | .1192012 | 0.536 | -.1597804 | .3074795 |
| -32 | .0719656 | .1192012 | 0.546 | -.1616644 | .3055955 |
| -31 | .034178 | .1181032 | 0.772 | -.1973 | .2656561 |
| -30 | .0138374 | .1181032 | 0.907 | -.2176406 | .2453155 |
| -29 | .0332814 | .1181032 | 0.778 | -.1981967 | .2647594 |
| -28 | .0238024 | .1181032 | 0.840 | -.2076756 | .2552805 |
| -27 | .0419811 | .1181032 | 0.722 | -.189497 | .2734591 |
| -26 | .079516 | .1191838 | 0.505 | -.1540799 | .3131119 |
| -25 | .1285722 | .1191838 | 0.281 | -.1050237 | .3621681 |
| -24 | .1626571 | .1191838 | 0.172 | -.0709388 | .396253 |
| -23 | .1962551 | .1191838 | 0.100 | -.0373408 | .429851 |
| -22 | .1921023 | .1191838 | 0.107 | -.0414936 | .4256982 |
| -21 | .2405146 | .1191838 | 0.044 | .0069187 | .4741105 |
| -20 | .2274367 | .1191838 | 0.056 | -.0061592 | .4610326 |
| -19 | .1773371 | .1181087 | 0.133 | -.0541516 | .4088258 |
| -18 | .1944296 | .1181087 | 0.100 | -.0370591 | .4259183 |
| -17 | .1979534 | .1181087 | 0.094 | -.0335353 | .4294421 |
| -16 | .2457385 | .1171236 | 0.036 | .0161806 | .4752965 |
| -15 | .2442794 | .1162133 | 0.036 | .0165055 | .4720533 |
| -14 | .2992883 | .1162133 | 0.010 | .0715145 | .5270622 |
| -13 | .3241741 | .1153466 | 0.005 | .0980989 | .5502494 |
| -12 | .3934798 | .1153466 | 0.001 | .1674046 | .6195551 |
| -11 | .4198394 | .1145746 | 0.000 | .1952772 | .6444015 |
| -10 | .3780205 | .1138593 | 0.001 | .1548604 | .6011806 |
| -9 | .3297838 | .1138593 | 0.004 | .1066237 | .5529439 |
| -8 | .3547956 | .1138593 | 0.002 | .1316355 | .5779557 |
| -7 | .3620875 | .1138593 | 0.001 | .1389274 | .5852476 |
| -6 | .3622198 | .1138593 | 0.001 | .1390597 | .5853799 |
| -5 | .3908874 | .1138593 | 0.001 | .1677273 | .6140474 |
| -4 | .3202365 | .1138593 | 0.005 | .0970765 | .5433966 |
| -3 | .1798646 | .1138593 | 0.114 | -.0432954 | .4030247 |
| -2 | -.0124409 | .1138593 | 0.913 | -.235601 | .2107192 |
| -1 | -.3400675 | .1146965 | 0.003 | -.5648685 | -.1152665 |
| Hour of death | -.9030667 | .1165413 | 0.000 | -1.131.483 | -.6746501 |
